# Enhancing the Transformer Model with a Convolutional Feature Extractor Block and Vector-Based Relative Position Embedding for Human Activity Recognition

**DOI:** 10.3390/s25020301

**Published:** 2025-01-07

**Authors:** Xin Guo, Young Kim, Xueli Ning, Se Dong Min

**Affiliations:** 1 Department of Software Convergence, Soonchunhyang University, Asan 31538, Republic of Korea; guoxinzhumengna@gmail.com (X.G.); n1461986424@gmail.com (X.N.); 2 Medical IT Engineering, Soonchunhyang University, Asan 31538, Republic of Korea

**Keywords:** human activity recognition, inertial measurement units (IMUs), transformer model, relative position embedding, convolutional neural networks (CNNs), time series signal

## Abstract

The Transformer model has received significant attention in Human Activity Recognition (HAR) due to its self-attention mechanism that captures long dependencies in time series. However, for Inertial Measurement Unit (IMU) sensor time-series signals, the Transformer model does not effectively utilize the a priori information of strong complex temporal correlations. Therefore, we proposed using multi-layer convolutional layers as a Convolutional Feature Extractor Block (CFEB). CFEB enables the Transformer model to leverage both local and global time series features for activity classification. Meanwhile, the absolute position embedding (APE) in existing Transformer models cannot accurately represent the distance relationship between individuals at different time points. To further explore positional correlations in temporal signals, this paper introduces the Vector-based Relative Position Embedding (vRPE), aiming to provide more relative temporal position information within sensor signals for the Transformer model. Combining these innovations, we conduct extensive experiments on three HAR benchmark datasets: KU-HAR, UniMiB SHAR, and USC-HAD. Experimental results demonstrate that our proposed enhancement scheme substantially elevates the performance of the Transformer model in HAR.

## 1. Introduction

**Figure 1:**
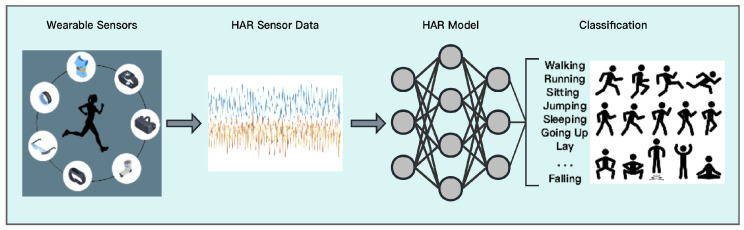
Example of a Human Activity Recognition system based on wearable sensors.

Human Activity Recognition (HAR) endeavors to comprehend human behaviors and states, utilizing AI systems to proactively furnish effective assistance based on human needs [1]. Consequently, HAR has evolved into a pivotal realm of investigation, playing an essential role in various domains, encompassing sports, healthcare, smart homes, abnormal activity detection, and smart driving [2,3,4,5,6]. The implementation of HAR in these domains typically relies on the utilization of cameras, WiFi-based transmitters and receivers, microphones, and Inertial Measurement Units (IMUs) for gathering data from humans. Nevertheless, cameras and microphones pose privacy concerns in numerous real-world application scenarios [7]. WiFi-based transmitters and receivers typically depend on channel state information, and the intricate nature of this model introduces challenges in signal acquisition, diminishing the model’s efficiency [8]. Consequently, smartphones and other wearable devices furnished with inertial sensors, like accelerometers and gyroscopes, find extensive application in HAR. This is attributed to their less intrusive monitoring of human activities and ease of wear. Illustrated in Figure 1, in general, HAR using IMUs sensors is a classification problem based on temporal signals, in which the corresponding sensor data are collected continuously in chronological order, and the activities are classified according to the user’s activities under different locations.

HAR focuses on analyzing sensor timing signals with long-term dependencies by applying feature extraction and pattern classification techniques to classify the activities performed by users [1,9]. In recent years, deep learning has emerged as the predominant technique in the HAR, automatically extracting high-level features tailored to the target task, thereby compensating for the absence of characterization in shallow, hand-crafted features typical in traditional machine learning. In particular, Convolutional Neural Networks (CNNs) [10] and their variants, such as ResNet [11], have garnered significant attention in recent times for leveraging the local dependencies and scale invariance present in sensor timing signals to improve the recognition capabilities of models [12]. Nevertheless, earlier studies [13] have shown that, for the classification of intricate actions, relying solely on the local dependencies of sensor timing signals is insufficient. This issue arises from the constrained receptive fields of CNN and their variants, making it challenging to extract global information from sensor timing data. Consequently, they struggle to effectively capture long dependencies in sensor timing signals [14]. Recurrent Neural Networks (RNNs) can leverage the correlation between neurons and are well-suited for addressing time-series problems. Specifically, RNN models utilizing Long Short-Term Memory (LSTM) can capture the temporal long dependencies in sensor time-series signals through LSTM units. However, the sequential nature of the recurrent architecture impedes the parallelization of model training, resulting in sluggish weight updates in the model network [15]. Moreover, its ability to model long dependencies is restricted by its limited memory capacity. On the other hand, the Transformer model [16], an architecture designed for highly parallel operations based on a self-attention mechanism, was proposed in previous research. It can capture long dependencies across the entire time series using the attention mechanism, and the extensive receptive domain of the attention mechanism provides richer contextual information [17]. Not surprisingly, given the success of the Transformer model in Natural Language Processing (NLP), numerous prior studies have explored its application in diverse domains, including computer vision [18], music series analysis [19], and others. Furthermore, the method has demonstrated superiority over RNNs and LSTM-based RNNs in sequence classification problems, achieving state-of-the-art generalization capabilities [20].

In the Transformer model, the self-attention mechanism serves as its core [16]. This attention mechanism, akin to human perception, seeks to selectively focus on specific regions of the target, enhancing fine details while suppressing irrelevant and potentially confusing information [21]. The self-attention mechanism is employed to calculate self-attention weights on the time series, capturing the temporal relationships within the input time series. However, a significant limitation of the self-attention mechanism is its divergence from a recurrent network structure, hindering its ability to effectively capture positional information along the time dimension [22]. This limitation becomes more pronounced in time series data due to its less informative data context [17]. Consequently, incorporating explicit representations of positional information becomes crucial, as proposed in methods like Sin-Cos Function Position Embedding. In contrast, prior HAR studies employing the Transformer model framework [15,20,23] have exclusively utilized APE (Sin-Cos Function). Nonetheless, Relative Position Embedding (RPE) offers an advantage by providing more positional information—specifically, information about the positions of different elements within the same sequence. This enhancement aids the model in capturing the interdependence of various positions and, consequently, in better comprehending the structural information of the time series [24], as demonstrated in [17]. However, for HAR, which is based on acceleration signals, further research and analyses are required.

To address the aforementioned challenges, this paper proposed a high-performance HAR model based on the Transformer architecture, drawing inspiration from the data processing approach in the Vision Transformer [25]. The model leverages the benefits of the convolutional layer for local dependencies and scale invariance. Recognizing that a sole convolutional layer falls short in preserving local contextual information, we employ three 1D convolutional layers as a feature extractor block to capture local features within the input time series. Subsequently, the Transformer’s self-attention mechanism is employed to adeptly capture the long dependencies within the input time series, utilizing both local and long features for activity classification. Simultaneously, to counteract the Transformer model’s potential oversight of position information within the input time-series data and to enrich the model with additional position information, this paper introduced a pioneering relative position embedding method, vRPE, specifically tailored for acceleration time-series signals associated with human activities. The primary contributions are as follows:

1. We proposed a novel Transformer model for IMU sensor-based HAR scenarios, which globally models the temporally localized features extracted by the convolutional network instead of the raw input signals, and more comprehensively takes into account the positional information within the sensor timing data via vRPE.
2. Within the realm of IMU sensor-based HAR, we conducted the first-ever verification using the Transformer model, demonstrating the superiority of RPE over APE. Furthermore, building upon the initial RPE, we introduce the vRPE method and validate its superiority over both existing APE and initial RPE.
3. To better utilize the different levels of features in the data, we proposed using a multi-layer convolutional neural network as a feature extractor to process the input data, which ensures that richer and hierarchical high-level features are built to improve the model’s generalization ability.
4. The model exhibits enhanced capability in comprehending the structural information of the input signal and representing features, surpassing the Baseline Models (BMs). Consequently, it attains superior classification performance on three publicly available human activity datasets, namely KU-HAR [26], UniMiB SHAR [27], and USC-HAD [28]. Moreover, the HAR model proposed in this paper has the best performance of our proposed model compared to the existing competitive HAR models.

The structure of this paper is as follows. Section 2 provides a review of related work. Section 3 details the proposed model, the baseline model, and the dataset information. Section 4 outlines the experimental setup and presents the results. Section 5 delves into the impact of the CFEB, Initial RPE, and our proposed relative position embedding on the Transformer model’s performance. Ablation experiments are conducted to scrutinize our model more comprehensively. Lastly, Section 6 concludes the paper.

## 2. Related Work

The core of IMU sensor-based HAR lies in modeling time-series signals with long-term dependencies [23]. Thus, extracting global information from sensor data enhances the efficacy of HAR and aids in comprehending intricate human activities. However, traditional machine learning methods like support vector machines, KNN, and ensemble learning in their early stages heavily depended on manually extracted shallow features [14]. These hand-crafted time-domain, frequency-domain, and time-frequency features [29] prove inadequate in capturing the inherent complexities of sensor time series signals, rendering them prone to errors, particularly in recognizing intricate activities such as excessive movements.

### 2.1. CNN- and RNN-Based Approaches

In recent years, deep learning, with its automatic feature extraction capability, has emerged as the predominant technique in HAR. In contrast to earlier IMU sensor-based HAR models, deep-learning-based HAR models can autonomously generate appropriate deep features through multiple internal hidden layers, thereby improving recognition performance. Numerous deep learning methods are constructed upon recurrent and convolutional architectures. CNN-based HAR models, leveraging their automatic feature representation capability, are extensively employed for extracting discriminative features from time series data, contributing to effective activity recognition. For instance, Veiga et al. [30] demonstrated the superior performance of CNN models over traditional machine learning models in both feature extraction and classification. In a previous study, Ronao et al. [31] developed a deep convolutional neural model by stacking multiple convolutional layers to extract more abstract high-level features from sensor time-series data. Concurrently, Ignatov et al. [32] introduced a network architecture that integrates local features extracted by CNNs with statistical features to capture global characteristics of sensor data. To automatically extract diverse local features and long-term dependency relationships from sensor time-series data using a CNN model, Zhang et al. [33] proposed a multiscale feature extraction fusion model that combines CNNs and Gated Recurrent Units (GRUs). Gao et al. [14] presented a selective convolutional kernel within a multi-branch CNN framework, Zhang et al. [10] introduced a multiple-head convolutional neural network with attention, and Tang et al. [34] employed hierarchical split convolution to generate multi-scale features. However, the feature representation capability of CNN-based HAR models depends on the number of convolutional layers or kernel size, limiting their ability to capture the long dependencies of sensor data along time series. Recurrent architectures, especially LSTM, are better suited to exploit the long dependencies of sensor signals for modeling. To enhance the capture of long dependencies in sensor data, Guan et al. [35] integrated multiple LSTM units, outperforming individual LSTM networks. Additionally, for effective utilization of both historical and subsequent temporal information in sensor data, Zhao et al. [36] developed a bidirectional LSTM framework. Alawneh et al. [37] compared unidirectional and bidirectional LSTM, demonstrating that bidirectional LSTM significantly outperform unidirectional LSTM, which utilize only historical temporal information. Furthermore, to overcome the limitation of CNN-based HAR models in modeling local information of the input data, Ordóñez et al. [13] proposed a DeepConvLSTM model that combines LSTM and CNN, outperforming CNNs alone. Nevertheless, in [38], several recurrent networks for HAR were analyzed, highlighting that a recurrent structure with sequential computation hinders the parallelization of sample training, leading to slow parameter updating and inference. Furthermore, LSTM is limited in its scope for modeling long dependencies because its memory units are prone to forgetfulness for earlier time points.

### 2.2. Transformer-Based Approach

Compared to RNNs and CNNs, attention mechanisms, which have emerged in recent years, demonstrate significant advantages in parallelism and modeling long dependencies. Consequently, attention mechanisms have garnered increasing attention from researchers and yielded promising results in the field of IMU sensor-based HAR. Particularly noteworthy is the Transformer model with a self-attention mechanism. In the initial applications within the NLP domain, the Transformer model [16] emerged as the most effective solution for sequential problems. Subsequently, with the success of advanced Transformer variants like Vision Transformer [26] and Swin Transformer [39] in the computer vision domain, the exploration of Transformer capabilities has gained significant attention in the field of HAR. Dirgová Luptáková et al. [40] discussed the superior performance of Transformer models in comparison to prior HAR approaches involving LSTM and CNNs. However, when applied to sensor time-series signals, the Transformer model fails to effectively leverage the a priori information regarding strong local correlations within the time series [17]. To address this limitation and efficiently capture the intricate dependencies and essential features of the sensor data, Tang et al. [15] introduced a two-branch Transformer framework incorporating a 2D convolutional layer. Additionally, Zhang et al. [23] proposed a Transformer model featuring an attention mechanism and two 1D convolutional layers. Meanwhile, in the realm of HAR, existing studies based on the Transformer model have overlooked the investigation of location coding within the Transformer model. A limitation of the core self-attention mechanism in the Transformer model is its lack of awareness regarding the location information of the input data. To address this limitation, practical strategies in transformer-based methodologies have been suggested, involving the use of various embedding techniques [41], such as APE and RPE, to enhance the temporal context of time-series inputs. For instance, Dosovitskiy et al. [25] introduced trainable APE. However, RPE, in contrast to APE, can capture more position information. Shaw et al. [22] proposed the initial RPE for self-attention. Nevertheless, this method requires computing attention weights by incorporating relative position information into both values and keys, introducing memory inefficiency [17]. In contrast to the aforementioned study, we introduce an effective Transformer model based on RPE for IMU sensor-based HAR scenarios. This model takes into account the relative position information inherent in the input acceleration timing signals, enabling the extraction of higher-level, stable local, and global features from the acceleration timing signal.

## 3. Methods

**Figure 2:**
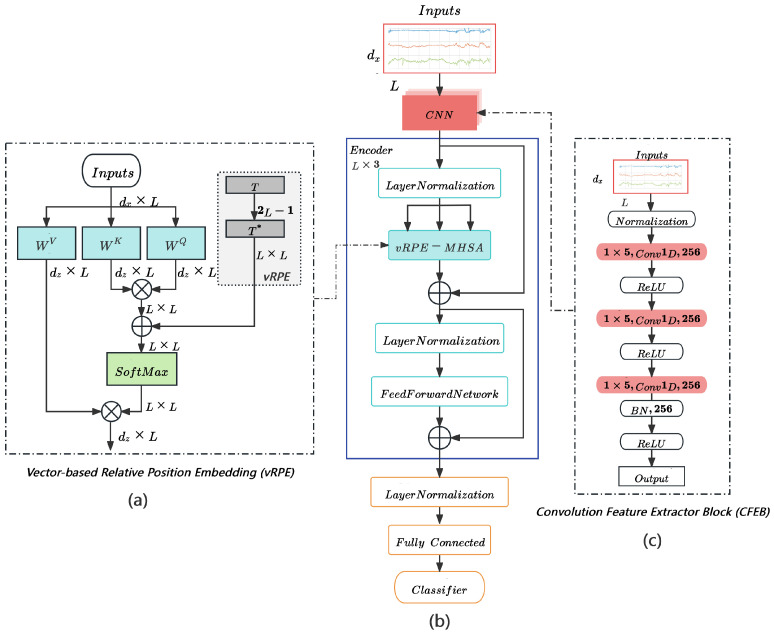
(**b**) is the overall model architecture. (**c**) is the dotted box on the right indicates the CFEB. Here, Normalization is the Z-Score standardized treatment and (**a**) is the Multi-head Self-Attention Mechanism with vRPE. Newly added parts are depicted in grey. Firstly, according to the relative position index, the extraction of the relative position value $T_{ij}^{*}$ is performed in *T* to obtain $T^{*}$. Furthermore, we provide $T_{ij}^{*}$ to $e_{ij}$ prior to SoftMax operations.

This paper aims to establish a high-performance HAR model based on the Transformer architecture. It encompasses two primary improvements. First, multi-layer convolutional layers capture local temporal features of acceleration signals. Second, we introduce vRPE to traditional MHSA, enabling the model to better interpret the structural information of sensor timing signals for stronger feature modeling. Figure 2 illustrates the model architecture. Initially, Z-score Normalization is applied to expedite training. Next, CFEB employs a fixed-size window with a one-dimensional convolutional layer to learn deeper local features in the temporal dimension. The encoder then extracts global temporal correlations in the time series using a multi-head attention mechanism with RPE, enhancing the model’s understanding of long dependencies. The final classification layer, comprising fully connected layers and a SoftMax function, produces classification outputs. Moreover, the hyperparameters of the model were optimized through ablation experiments. Ultimately, end-to-end training on publicly available datasets produced a model that outperformed existing methods. The following sections provide further model details.

### 3.1. Inputs Definition

The given time series dataset X consists of *n* samples, $X=\{X_{1},X_{2},\ldots ,X_{n}\}$, where $X_{t}=\left\{x_{1},x_{2},\ldots ,x_{L}\right\}$ represents a $d_{x}$-dimensional time series with length *L*. Here, $X_{t}\in R^{L\times d_{x}}$, and the set of corresponding response labels is denoted as $Y=\{y_{1},y_{2},\ldots ,y_{n}\}$, where $y_{t}\in \left\{1,\ldots ,c\right\}$ and *c* signifies the number of classes.

### 3.2. Convolutional Feature Extractor Block

IMU-based HAR tasks usually involve time-series data from sensors, which have significant local time dependence [15,23]. Due to the excellent local awareness and translation invariance properties of convolutional layers, they can effectively extract local time-dependent features in time series and improve generalization performance [42]. However, single-layer convolutional layers are deficient in preserving contextual information, and especially, they have limited effectiveness in modeling patterns across time windows. Therefore, we design CFEB to capture multi-scale local time-dependent features by stacking three 1D convolutional layers. This design not only strikes a balance between computational efficiency and classification performance, but also more fully extracts the local contextual information in the signal, where each layer utilizes an identical set of 256 filters and maintains a consistent kernel size of 5. Meanwhile, to reduce the computational overhead of CFEB as well as to improve the generalization performance of the whole model, we will add the batch normalization layer after the last convolutional layer. Therefore, for a given input sample $X_{t}=\left\{x_{1},x_{2},\ldots ,x_{L}\right\}$, local temporal feature extraction is conducted through convolutional layers, yielding convolutional output $F_{j}\in R^{L\times D}$ (where *D* represents the mapping dimension of the convolutional layer) for j=1,2,3. The CFEB, depicted in the dashed box on the right side of Figure 2c, specifically processes $X_{t}$ through a Convolution Feature Extractor Block that comprises a normalization layer, 1D convolutional layers, batch normalization layer, and ReLU activation. This process is expressed as:

(1) $$F_{1}=\mathrm{ReLU}({\mathrm{Conv}1D}_{1}\left(\mathrm{Normalization}\left(X_{t}\right)\right));$$

(2) $$F_{2}=\mathrm{ReLU}({\mathrm{Conv}1D}_{2}\left(F_{1}\right));$$

(3) $$F_{3}=\mathrm{ReLU}(\mathrm{BN}\left({\mathrm{Conv}1D}_{3}\left(F_{2}\right)\right)).$$

### 3.3. Multi-Head Self Attention

The attention mechanism can be characterized as a process that maps a query and a set of key-value pairs to an output. This output is computed as a weighted sum of values, with the weight assigned to each value determined by the compatibility function of the query with the corresponding key [16]. Initially, attention mechanisms were introduced within the domain of natural language processing, relying on recurrent neural networks at their core [43]. However, Vaswani et al. [16] proposed a transformer model that exclusively utilizes self-attention. In this model, a query and a set of key-value pairs are mapped to an output. The query vector represents the current element in the sequence, while the key and value vectors represent the other elements in the sequence. To be more specific, for an input sequence, $X_{t}=\left\{x_{1},x_{2},\ldots ,x_{L}\right\}$, self-attention computes an output sequence $Z_{t}=\left\{z_{1},z_{2},\ldots ,z_{L}\right\}$, where $z_{i}\in R^{d_{z}}$. This output is calculated as a weighted sum of input elements:

(4) $$z_{i}=\sum_{j=1}^{L}\alpha_{i,j}\left(x_{j}W^{V}\right).$$

Each coefficient weight $\alpha_{i,j}$ is calculated by using the SoftMax function:

(5) $$\alpha_{i,j}=\frac{exp\left(e_{ij}\right)}{\sum_{k=1}^{L}exp\left(e_{ik}\right)}.$$

where $e_{ij}$ is an attention weight from positions *j* to *i* and is computed using a scaled dot-product:

(6) $$e_{ij}=\frac{\left(x_{i}W^{Q}\right){\left(x_{j}W^{K}\right)}^{T}}{\sqrt{d_{z}}}.$$

The projection matrices $W^{Q},W^{K},\;\mathrm{and}\;W^{V}\in R^{d_{x}\times d_{z}}$ are individual parameter matrices unique to each layer. Instead of performing self-attention just once, the Multi-Head Self-Attention (MHSA) [16] approach carries out this process in parallel multiple times, employing *h* attention heads. The output from each attention head undergoes a linear transformation and is then concatenated to conform to the standard dimensions, namely:

$$\mathrm{MHSA}\left(Q,K,V\right)=\mathrm{Concat}\left({\mathrm{head}}_{1},\ldots ,{\mathrm{head}}_{h}\right)W^{O},$$

(7) $${\mathrm{head}}_{i}=\mathrm{Self}\;\mathrm{Attention}\left(X_{t}W_{i}^{Q},X_{t}W_{i}^{K},X_{t}W_{i}^{V}\right).$$

where $W^{o}\in R^{hd_{z}\times L}$, $Q=X_{t}W_{i}^{Q}$, $K=X_{t}W_{i}^{K}$, $V=X_{t}W_{i}^{V}$.

### 3.4. Vector-Based RPE

The Transformer architecture relies on explicit positional encoding to retain the positional information of the input data [41]. Consequently, self-attention alone is incapable of capturing positional information along the time dimension. This inherent limitation is further pronounced when applied to time series data, as such data inherently possess less contextual information compared to other data types [17]. To address this limitation, in this paper, a method is proposed that utilizes the following RPE approach. This approach primarily involves incorporating the relative positional bias $T_{ij}^{*}$ into each head when calculating weights in the self-attention mechanism. Adding relative positional bias $T_{ij}^{*}$ to (6):

(8) $$e_{ij}=\frac{\left(x_{i}W^{Q}\right){\left(x_{j}W^{K}\right)}^{T}}{\sqrt{d_{z}}}+T_{ij}^{*}.$$

The vRPE we propose is an extension of the attention mechanism, specifically designed to consider the pairwise relationship between two-time points within the input timing signal. The algorithm is presented in Algorithm 1. In Equation (8), the relative position bias $T_{ij}^{*}$ corresponds to the value at position (i,j) in vector $T^{*}$. The relative position indexTable $I\in R^{L\times L}$ is computed from the positional differences between each time point and other time points in $X_{t}$ using a fixed window size. Subsequently, the relative position vector $T^{*}\in R^{L\times L}$ is obtained by using the index value in the *I* to fetch the value in the trainable relative position biasTable $T\in R^{2L-1}$. Accordingly, we need to index $L^{2}$ elements from the 2L−1 vector. The flowchart of the self-injecting attention mechanism operation with vRPE, as can be seen in Figure 2a. Furthermore, as shown in Table 1, our proposed vRPE is more efficient in terms of both memory and time complexity compared to the initial RPE methods in the literature.
**Algorithm 1** Vector-based Relative Position Embedding  1: **Input:**   2:     $X_{t}\in R^{L\times d}$▹ Input sequence of length *L* and dimension *d*  3:     $W^{Q},W^{K}\in R^{d\times d_{z}}$▹ Query and Key weight matrices  4:     $T\in R^{2L-1}$▹ Trainable relative position bias vector  5:     $I\in R^{L\times L}$▹ Relative position index table  6: **Output:**   7:     $e_{ij}\in R^{L\times L}$▹ Relative position bias added to attention weights  8: **Initialization:**   9:     Compute the relative position index table *I* based on positional differences10:     Fetch the relative position bias vector $T^{*}\in R^{L\times L}$ using *I* and *T*11: **for** each time point *i* from 1 to *L* **do**12:       **for** each time point *j* from 1 to *L* **do**13:             Compute the query vector $Q_{i}=X_{i}W^{Q}$14:             Compute the key vector $K_{j}=X_{j}W^{K}$15:             Compute the dot product $Q_{i}K_{j}^{T}$ for self-attention score16:             Add the relative position bias $T_{ij}^{*}$ from $T^{*}$ to the self-attention score17:             Calculate the final attention score: $e_{ij}$18:       **end for**19: **end for**20: **Return:** 
$e_{ij}$

### 3.5. Feed-Forward Network

The Feed-Forward Network (FFN) alters the output space of the Multi-Head Self-Attention, introducing non-linearity to the Transformer model. The FFN consists of two fully connected layers with a GeLU activation function in between. The corresponding formula is as follows:

(9) $$FFN\left(x\right)=GeLU\left(xW_{1}+b_{1}\right)W_{2}+b_{2}.$$

### 3.6. Baseline Models

CNNs model [31]: As a representative deep learning method, CNNs has become a powerful benchmark. We adapted a one-dimensional CNN training scheme based on [31] for the three HAR datasets utilized in the experiments. The model comprises three convolutional layers, each connected by a Max Pooling layer activated by a ReLU non-linear function. The output features of the final layer are mapped to a fully connected layer where a Softmax operation is subsequently applied to predict the corresponding probabilities for each activity category.

CNNs-LSTM model [13]: The advantages of deep convolutional and recurrent networks in sequence modeling tasks have been extensively investigated. For comparison purposes, we replicate the widely used CNNs-LSTM framework [13] on three benchmark datasets for HAR. The framework is designed by stacking three 2D convolutional layers, each followed by a BatchNorm layer. Two LSTM layers are then inserted to capture long-range dependencies. The filters for the three convolutional layers are 64, 128, and 256, respectively, and the activation function is ReLU. Finally, the output of the third convolutional layer is fed into two LSTM layers, each with 128 hidden neurons. The output of the second LSTM layer is flattened and fed into a fully connected layer to produce the final Softmax probability for activity recognition.

Transformer model [40]: The self-attention mechanism in the Transformer model calculates potential correlations among the temporal signals themselves in parallel to capture long-range dependencies [15]. Therefore, to assess the relative performance gains resulting from the proposed approach, the Transformer encoder model [40] is employed as the BM in this paper. The BM comprises three main parts. The first part is the position embedding section, utilizing APE [16]. The second part consists of the Transformer encoder layers. In this paper, three encoder layers are configured, with each layer having an input dimension $d_{\mathrm{input}}$ of 256, a multi-head self-attention *n-heads* of 8, and a feed-forward neural network dimension $d_{\mathrm{ff}}$ of 256.

### 3.7. Dataset Description and Preprocessing

**Figure 3:**
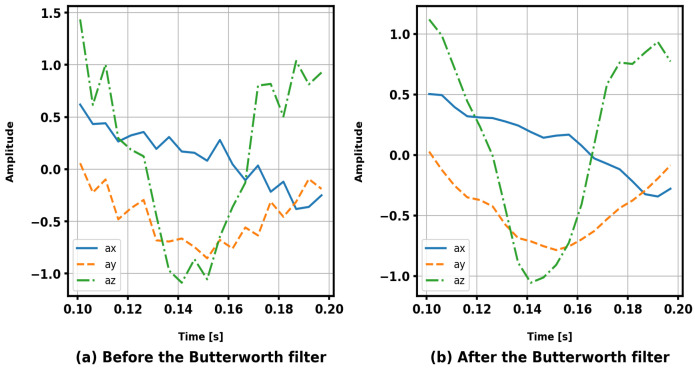
This figure illustrates a segment of activity data randomly selected from the UniMiB SHAR dataset and its corresponding filtered result. Subfigures (**a**,**b**) represent the signal waveforms before and after filtering, respectively. The comparison demonstrates that the Butterworth filter not only effectively smooths the signal but also preserves critical low-frequency features, making it highly suitable for applications in human activity recognition.

In this study, we conducted experiments using three widely used public datasets: KU-HAR [26], UniMiB SHAR [27], and USC-HAD [28]. These datasets offer a diverse range of activity categories and intricate data distributions, providing robust evidence for evaluating HAR systems. This paper selected three publicly available human activity datasets for evaluation and adopted various data preprocessing methods to improve activity recognition accuracy. Specifically, a third-order Butterworth low-pass filter with a cutoff frequency of 20 Hz is suitable for noise reduction, as nearly all measurable body motion is confined to frequency components below 20 Hz, with 99% of the signal energy concentrated below 15 Hz [44], thus not resulting in the loss of important features in the data, as shown in Figure 3. Then, a low-pass filter with a cut-off frequency of 0.2 Hz was employed to eliminate the effect of gravity [11,27]. Sensor data were segmented into continuous samples using a sliding window technique. Window size significantly influences the performance of activity recognition. According to a previous study [45], due to the diversity of human activities in the datasets, there is no clear consensus on the optimal window size. Therefore, to ensure a fair comparison, we adopted the same parameter settings used in [23,26,27]. We summarize the key details of the three datasets in Table 2.

## 4. Experiments

In this section, we initially provide essential details about the the evaluation metrics utilized to gauge model performance. Subsequently, we outline the pertinent experimental setup and model training specifics. Finally, we delve into the description of the experimental results, featuring comparative analyses with various state-of-the-art models proposed in recent literature, alongside the BMs.

### 4.1. Evaluation Metrics

The performance evaluation of deep learning models depends on various metrics. Moreover, the class imbalance of data categories is prevalent when gathering information on human behavior in the environment. For this reason, we adopt an average F1-score and Accuracy as the performance metric to evaluate HAR [46], with the former representing the reconciled average of precision and recall, and the latter representing the overall accuracy across all categories, mathematically formulated as:

(10) $$Accuracy_{i}=\frac{1}{M}\sum_{i=1}^{M}\frac{{TP}_{i}+{\mathrm{TN}}_{i}}{{TP}_{i}+{TN}_{i}+{FP}_{i}+{FN}_{i}}.$$

(11) $$F_{1}\text{-}\mathrm{Score}\;=\frac{1}{M}\sum_{i=1}^{M}\frac{2\times Precision_{i}\times Recall_{i}}{Precision_{i}+Recall_{i}}.$$

where *M* denotes the number of total classes and ${TP}_{i}$, ${FP}_{i}$, ${TN}_{i}$, and ${FN}_{i}$, respectively, represent true positives, false positives, true negatives, and false negatives in the *i*-th activity class.

### 4.2. Experimental Setup Details

To ensure effective generalization capability, we divided the KU-HAR and UniMiB SHAR datasets into training (70%), validation (10%), and test (20%) sets, as suggested by previous studies [1,15,21]. For the USC-HAD dataset, subjects 1–10 were used for training, subjects 11 and 12 for validation, and the rest for testing [23]. Therefore, both the baseline and proposed models were evaluated using the same preprocessed datasets, which include consistent dataset partitioning (training, testing, and validation sets), window size, overlap rate, and batch size. Detailed model settings are presented in Table 3. We employed label smoothing regularization to handle potentially mislabeled data and prevent overfitting, using a smoothing factor of 0.1 [15]. Cross-entropy loss was used to optimize model weights during training. The Adam optimizer updated the weights to minimize the cross-entropy loss function. An adaptive learning rate adjustment strategy, starting with an initial rate of 0.001, was used to improve convergence based on accuracy. To enhance training quality and generalization, we applied Dropout regularization to both the FFN and vPRE-MHSA components in the encoder layer. Additionally, Layer Normalization was performed on the inputs to both components to boost training efficiency. Throughout the experiments, the maximum training epoch was set to 50. An early stopping mechanism was implemented to halt training if performance did not improve within a specified epoch range, thereby avoiding over-fitting [31]. The model with the best validation performance was saved for testing.

All programs were implemented using TensorFlow 2.5 with Python 3.8 on a desktop server equipped with an AMD Ryzen 9 5900X 12-Core Processor, NVIDIA RTX 3090 GPU, and 64 GB of RAM.

### 4.3. Experimental Results

**Figure 4:**
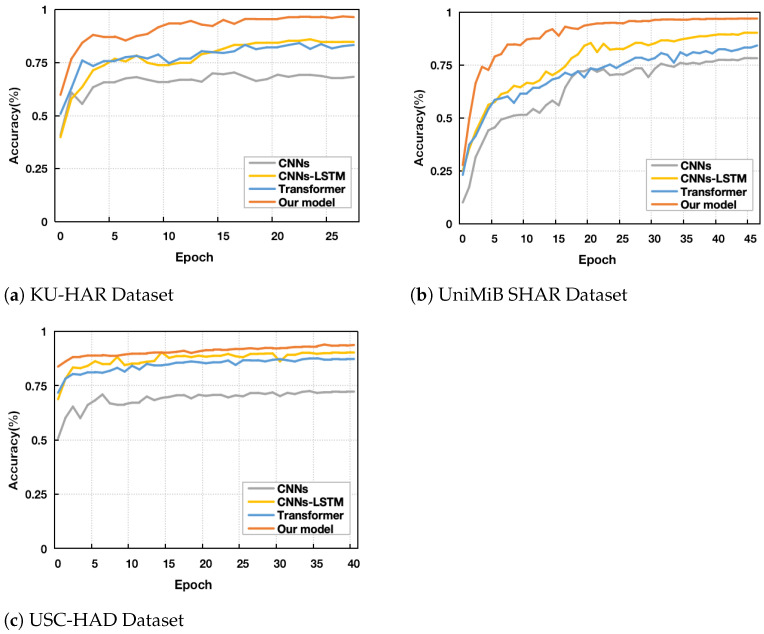
Validation Accuracy curves for the models on three datasets.

One of the primary goals of this study is to investigate the influence of combining CFEB, vRPE, and the baseline model Transformer on the classification performance of human activities. Due to sample imbalance in all three datasets used for model evaluation, a thorough assessment of the model’s performance is crucial, as shown in Table 4. Firstly, Table 5 presents a comparison of the F1 scores and Accuracy between our proposed model and the baseline methods across three benchmark HAR datasets. Figure 4 illustrates the validation accuracy curves of the various models. It is clear that on the UniMiB SHAR dataset, our model achieves the remarkable performance, with an accuracy of 97.20%, significantly surpassing other baseline methods. On the USC-HAD dataset, our model again exhibits excellent results, with an F1-score of 92.70% and an accuracy of 94.10%. On the KU-HAR dataset, despite the increased number of activity categories, our model demonstrates outstanding performance, achieving an accuracy of 96.80% and an F1 score of 97.50%, further validating its robustness and generalization ability. Notably, on the KU-HAR dataset, our model achieves F1 score improvements of 29.40%, 8.70%, and 10.10% compared to CNNs, CNNs-LSTM, and Transformer, respectively. This performance advantage is consistently observed across other datasets as well. In contrast, the CNNs and Transformer models underperform on all three datasets due to their limitations in local feature extraction and loss of detailed activity information. Additionally, while the hybrid model CNNs-LSTM achieves relatively better performance by combining local representations with long-term dependencies, it still falls short of our proposed model. We also noticed performance variations across datasets for the same model, which could be attributed to differences in data quality, such as annotation devices, sampling rates, and sample distributions. In addition, to further evaluate the performance of the proposed model, we also compare the proposed model with a series of highly competitive approaches in the previous literature, including [23,47,48,49,50,51,52,53,54,55,56,57], and the main results are summarized in Table 5. These studies used the same dataset to validate their model algorithms, but the data preprocessing methods varied. Therefore, the comparison results are not absolute, but provide relative insights into model performance.

## 5. Discussion

In this subsection, we first focus on the impact of CFEB and vRPE on enhancing the Transformer (TF) model. Illustrated in Table 6, the enhancement methods proposed in this study for the Transformer model exhibit significant efficacy, elevating both the generalization ability and activity classification performance to a higher level. Consequently, a more detailed explanation is warranted to elucidate how these improvement methods enhance the generalization ability and classification performance of the Transformer model. Secondly, we will perform a series of ablation studies to analyze our model structure more comprehensively, specifically investigating the impact of various hyper-parameters such as the number of convolution layers, the filter size of the convolutional layers, and the number of heads in the multi-head attention mechanism on the performance of activity classification.

### 5.1. Effect of Improved Methods

**Figure 5:**
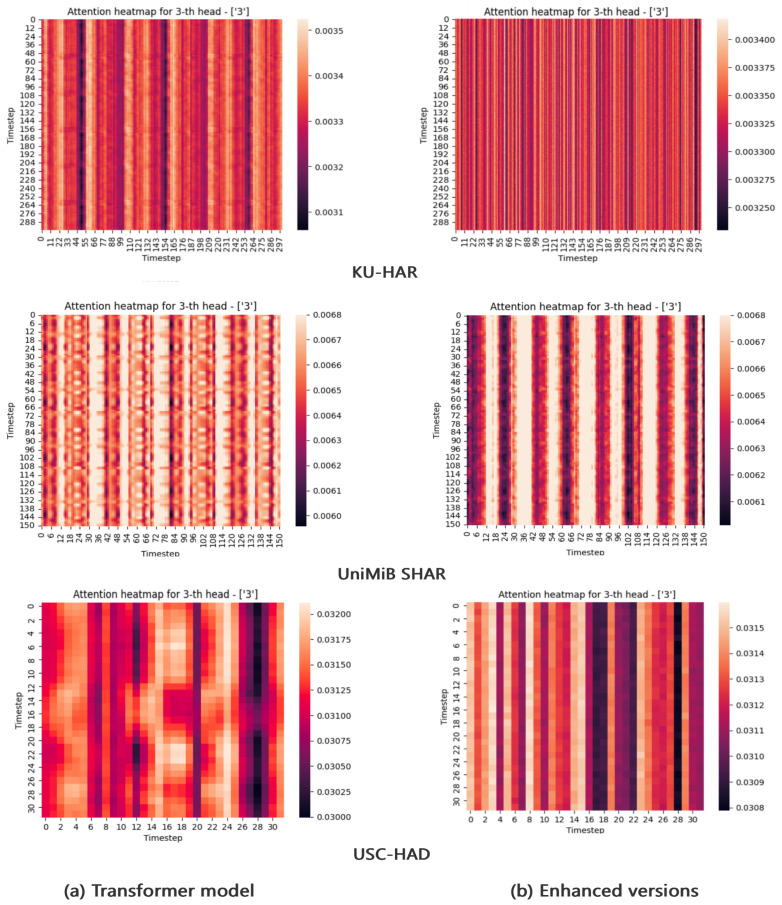
Attention scores visualization of Transformer model (**a**) and enhanced versions (**b**). By using the CFEB module, the local features of the acceleration signal are learned, which further enhances the attention weights of the Multi-Head self-attention mechanism layer.

*Effect of CFEB*: It is well known that the classification performance of HAR models based on acceleration signals relies heavily on the analysis of input data, both in traditional machine learning and deep learning [23]. Therefore, we explore the effect of CFEB on the self-attention mechanism when it analyzes the input data. Firstly, based on the classification results presented in Table 6, it is evident that the Transformer model’s proficiency in recognizing human activities has improved. Subsequently, to enhance the illustration of CFEB’s impact on weight calculation, attention mechanism heatmaps were generated. As shown in Figure 5, where Figure 4a represents the Transformer model’s 3rd head’s attention scores heatmap, and Figure 4b denotes the Transformer model’s improved 3rd head’s attention scores heatmap using the CFEB. Lighter shades in the heatmaps signify higher attention weights, with the horizontal and vertical coordinates denoting the timestep. Clear observation reveals that Figure 4a displays a more evident regular structure compared to Figure 4b, suggesting that CFEB has effectively captured specific time scale information from acceleration data and mitigated the impact of acceleration data noise. Consequently, the attention mechanism is more inclined to concentrate on the localized characteristics of the useful time scale extracted by the CFEB when computing attention weights between each timestep and others.

**Figure 6:**
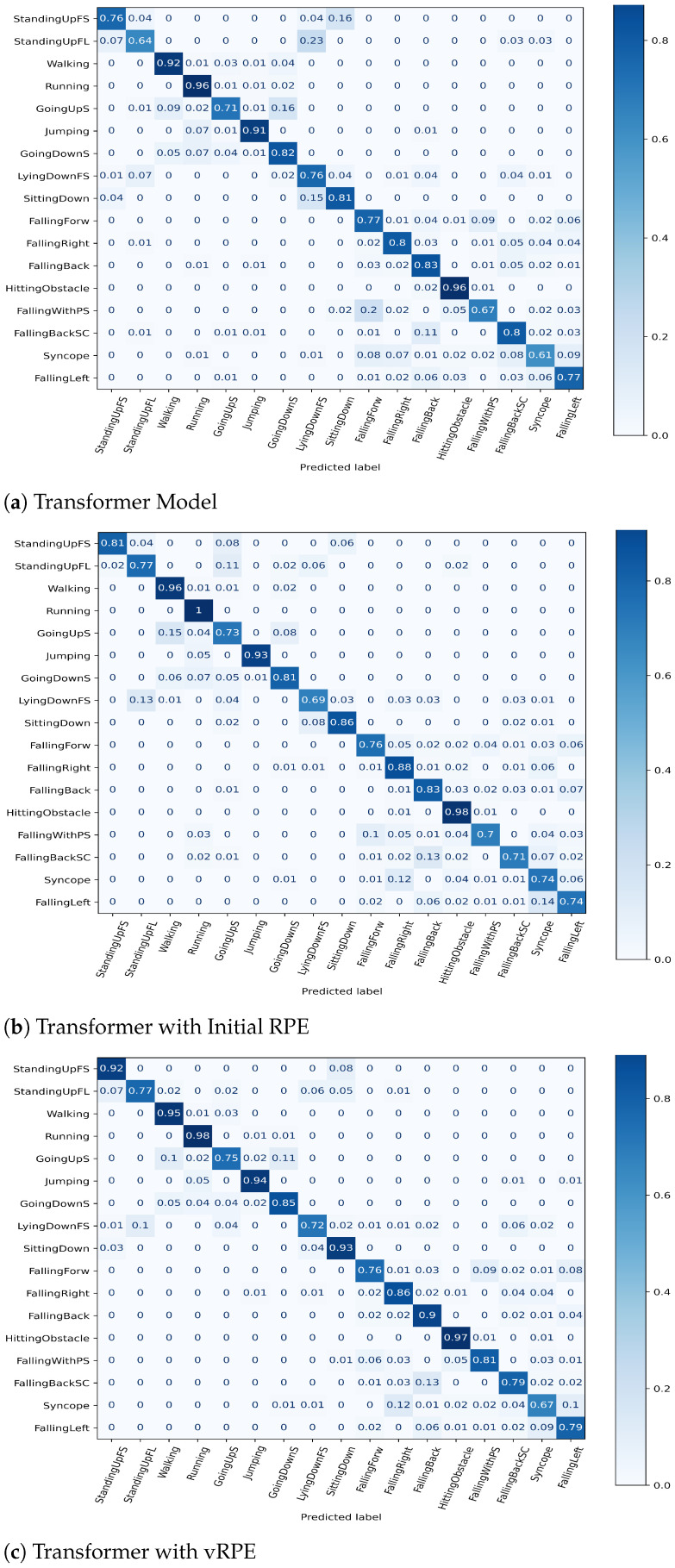
The Confusion Matrix for UniMiB SHAR dataset.

*Effect of vRPE*: We demonstrated on the UniMiB SHAR dataset that RPE can enhance the classification performance of the Transformer model in sensor-based activity classification tasks. Furthermore, it was verified that vRPE can further improve the performance of the Transformer model. Additionally, we compared the classification performance of the baseline Transformer model under different position embedding schemes, including APE [16], initial RPE [22], and proposed vRPE. The results presented in Table 7 demonstrate that the use of initial RPE improves Accuracy and F1-Score by 2.10% and 3.20%, respectively, when compared to APE. To some extent, this validates our hypothesis. Utilizing the Transformer model, the RPE approach proves effective not only in CV and NLP but also yields positive outcomes in handling HAR acceleration signals. Simultaneously, the introduced vRPE enhances the Accuracy and F1-Score of the baseline Transformer model by 1.50% and 1.70%, respectively, relative to the Initial RPE approach. To further illustrate the effectiveness of the proposed RPE, we visualize the confusion matrix of the baseline Transformer model under three different position embedding conditions. According to the confusion matrix plots in Figure 6a–c, it is evident that the baseline model with vRPE shows improved performance in classifying excessive actions, specifically in classes such as “StandingUpFS”, “StandingUpFL”, “SittingDown”, and “GoingUps”. Furthermore, the classification of excessive movements (motion transitions) relies more heavily on the model’s fine-grained perception of the input data [58]. Therefore, vRPE helps the model better understand the temporal distances and correlation information between different time steps, thereby enhancing its ability to perceive positional relationships.

### 5.2. Ablation Work

Impact of Convolutional Layer Number: In theory, a higher number of layers enhances the model’s capacity to capture local features of the acceleration signal. However, it also increases the risk of over-fitting, necessitating a balance [59], which aligns with our experimental observations. In this study, the number of convolutional layers is increased sequentially from one layer where the number of convolutional kernels per layer is 256 other parameters are kept constant, we present the classification results in Table 8. Notably, the model achieves superior performance when the number of layers is set to 3. Subsequently increasing the number of layers leads to a decline in classification performance. Therefore, this indicates that the CFEB, with its three-layer convolutional structure, can effectively capture the multi-scale local temporal features of HAR data, addressing the limitations of single-layer convolution.

Impact of Convolutional Filter Numbers: In our proposed model framework, the CFEB comprises multiple convolution layers. The number of filters in the convolution layers directly impacts their ability to extract local features. However, blindly increasing the number of filters can have adverse effects on both the model’s classification performance and lead to over-fitting [60,61]. As mentioned previously, we maintain uniform filter counts across all convolutional layers in the CFEB. While keeping the other hyper-parameters unchanged, we incrementally raise the number of filters for each convolutional layer within the CFEB. Furthermore, as indicated in the classification results within Table 9, the model achieves its highest Accuracy and F1-Score when the filter count is 256. Hence, we opted to set the filter count to 256 for each convolutional layer in this study.

Impact of Head Number: In this study, the number of heads is also a hyper-parameter that enhances the model’s classification ability by enabling it to focus on different information of the input in distinct representation subspaces [62]. As demonstrated in Table 10 for the activity classification results, the highest classification accuracy and F1-Score values are observed when the number of heads in the multi-head attention mechanism is set to 8, signifying optimal performance for the proposed model at this configuration. Therefore, we employed 8 heads in this study.

## 6. Conclusions

This paper utilizes the Transformer model to capture global information from acceleration time series signals and explores enhancements for robustness in IMU sensor-based activity recognition. Initially, we incorporated RPE into the conventional MHSA mechanism, introducing relative timing position information to enhance the Transformer’s ability to model acceleration signals. Building on this foundation, we proposed the vRPE method, which proved more effective than APE and the initial RPE in helping the Transformer model understand distance and correlation information present in acceleration data. This improves the model’s fine-grained perception of actions, particularly the classification of excessive actions. Additionally, we addressed the Transformer’s limitation in capturing local features by introducing the CFEB, which incorporates local dependencies and scale invariance. Experimental results showed that CFEB improved the Transformer’s performance in analyzing action signals. Finally, testing on several benchmark public HAR datasets demonstrated that our proposed methods enhance the Transformer model’s robustness for HAR tasks.

Future Research: The experimental results demonstrate the proposed model’s effectiveness in accurately classifying activity categories within IMU sensor time series signals. However, further research is needed for real-world applications, as sensor placement and activity variability across different age groups may cause model instability. Our future work will focus on developing and testing practical HAR applications to ensure the model’s effectiveness and applicability in real-world scenarios.

## Figures and Tables

**Table 1:** Comparing the parameter sizes, memory, and computation complexities of various position encoding methods.

Method	Parameter	Memory	Complexity
Shaw [22]	$\left(2L-1\right)d_{z}$	$L^{2}d_{z}+L^{2}$	$L^{2}d_{z}$
vRPE	2L−1	$L+L^{2}$	$L^{2}$

**Table 2:** Main information of the datasets.

	KU-HAR [26]	UniMiB SHAR [27]	USC-HAD [28]
Number of Subjects	90	30	14
Categories	18	17	12
Sensor Locations	Waist	Sweatpants front pockets	Right hip
Sensor Channels	6	3	6
Sensor	Samsung Galaxy J7	Samsung Galaxy Nexus I9250	MotionNode
Sample Rate	100 Hz	50 Hz	100 Hz
Window Size	300 × 1	151 × 1	32 × 1
Overlap Rate	-	50%	50%
Batch Size	64	64	256

**Table 3:** Experimental setup details.

	KU-HAR	UniMiB SHAR	USC-HAD
Maximum Epochs	50	50	50
Initial Learning Rate	0.001	0.001	0.001
Dropout	0.2	0.2	0.2
Smoothing Factor	0.1	0.1	0.1
Layers of Encoder	3	3	3
FFN Size	256	256	256

**Table 4:** Performance of the our model on three datasets.

Dataset and Evaluation	Accuracy	F1-Score	Precision	Recall
KU-HAR	96.80%	97.50%	97.50%	97.51%
UniMiB SHAR	97.20%	94.90%	94.90%	94.51%
USC-HAD	94.10%	92.70%	92.70%	92.60%

**Table 5:** The performance comparisons on HAR datasets. The symbol “-” denotes no results.

Methods and Performance		KU-HAR	UniMiB SHAR	USC-HAD
		Accuracy and F1-Score		
BMs	*CNNs*	67.70% & 68.10%	78.20% & 78.90%	72.70% & 70.90%
	CNNs-LSTM	84.90% & 88.80%	89.50% & 86.40%	90.90% & 89.70%
	Transformer	83.20% & 87.40%	83.70% & 79.70%	87.90% & 83.40%
Others’ Research		88.53% & - [47]	80.33% & 79.89% [48]	86.00% & 86.00% [23]
		- & 94.25% [49]	88.63% & - [50]	88.32% & 88.25% [51]
		94.76% & 94.73% [52]	93.90% & - [53]	91.25% & - [54]
		95.46% & 95.34% [55]	95.27% & - [56]	91.70% & - [57]
Our Model		96.80% & 97.50%	97.20% & 94.90%	94.10% & 92.70%

**Table 6:** Experimental test result for different model modules on three datasets.

Module	KU-HAR	UniMiB SHAR	USC-HAD
	Accuracy and F1-Score		
TF (BM)	83.20% & 87.40%	83.70% & 79.70%	87.90% & 83.40%
TF + vRPE	91.20% & 93.10%	87.30% & 84.60%	90.90% & 88.80%
TF + CFEB	95.70% & 95.80%	95.30% & 91.80%	92.00% & 90.00%
TF + vRPE + CFEB	96.80% & 97.50%	97.20% & 94.90%	94.10% & 92.70%

**Table 7:** Experimental test result of different position embedding methods on UniMiB SHAR dataset.

Model Variation	Accuracy	F1-Score
Vaswani [16]	83.70%	79.70%
Shaw [22]	85.80%	82.90%
vRPE	87.30%	84.60%

**Table 8:** Our model Accuracy and F1-Score for different Convolutional layer number on three datasets.

Layer Number	KU-HAR	UniMiB SHAR	USC-HAD
	Accuracy and F1-Score		
1	86.80% & 90.60%	80.20% & 90.70%	90.60% & 91.40%
2	95.50% & 97.30%	92.00% & 94.20%	91.60% & 92.30%
3	96.80% & 97.50%	97.20% & 94.90%	94.10% & 92.70%
4	96.00% & 96.40%	97.00% & 94.70%	93.50% & 91.90%
5	95.70% & 96.50%	96.80% & 94.50%	93.10% & 90.40%

**Table 9:** Our model Accuracy and F1-Score for different Convolutional filter numbers on three datasets.

Filter Number	KU-HAR	UniMiB SHAR	USC-HAD
	Accuracy and F1-Score		
64	94.60% & 95.40%	94.80% & 91.30%	91.10% & 88.90%
128	96.10% & 96.80%	95.10% & 91.50%	92.70% & 89.90%
256	96.80% & 97.50%	97.20% & 94.90%	94.10% & 92.70%
512	90.60% & 90.10%	89.00% & 80.00%	89.50% & 86.50%

**Table 10:** Our model Accuracy and F1-score for different head counts on three datasets.

Head Number	KU-HAR	UniMiB SHAR	USC-HAD
	Accuracy and F1-Score		
2	94.50% & 95.20%	94.00% & 90.20%	90.70% & 89.20%
4	95.50% & 96.50%	93.00% & 88.30%	92.00% & 89.70%
8	96.80% & 97.50%	97.20% & 94.90%	94.10% & 92.70%
16	96.10% & 97.00%	95.70% & 92.40%	91.70% & 89.50%

## Data Availability

Data are contained within the article.

## References

[1] Kim E. (2020). Interpretable and Accurate Convolutional Neural Networks for Human Activity Recognition. IEEE Trans. Ind. Inform..

[2] Xiao Z., Yu F., Liu L., Peng T., Hu X., Jiang M. (2024). DSANet: A lightweight hybrid network for human action recognition in virtual sports. Comput. Animat. Virtual Worlds.

[3] Shah S.H.H., Karlsen A.S.T., Solberg M., Hameed I.A. (2024). An efficient and lightweight multiperson activity recognition framework for robot-assisted healthcare applications. Expert Syst. Appl..

[4] Abuhoureyah F.S., Wong Y.C., Mohd Isira A.S.B. (2024). WiFi-based human activity recognition through wall using deep learning. Eng. Appl. Artif. Intell..

[5] Fan J., Liu Z., Du H., Kang J., Niyato D., Lam K.Y. (2024). Improving Security in IoT-Based Human Activity Recognition: A Correlation-Based Anomaly Detection Approach. IEEE Internet Things J..

[6] Li T., Li X., Ren B., Guo G. (2024). An Effective Multi-Scale Framework for Driver Behavior Recognition With Incomplete Skeletons. IEEE Trans. Veh. Technol..

[7] Qian H., Pan S.J., Miao C. (2021). Weakly-supervised sensor-based activity segmentation and recognition via learning from distributions. Artif. Intell..

[8] Akhtar Z.U.A., Wang H. (2020). WiFi-Based Driver’s Activity Monitoring with Efficient Computation of Radio-Image Features. Sensors.

[9] Hernandez V., Dadkhah D., Babakeshizadeh V., Kulić D. (2021). Lower body kinematics estimation from wearable sensors for walking and running: A deep learning approach. Gait Posture.

[10] Zhang H., Xiao Z., Wang J., Li F., Szczerbicki E. (2019). A novel IoT-perceptive human activity recognition (HAR) approach using multihead convolutional attention. IEEE Internet Things J..

[11] Xu S., Zhang L., Huang W., Wu H., Song A. (2022). Deformable convolutional networks for multimodal human activity recognition using wearable sensors. IEEE Trans. Instrum. Meas..

[12] Wang J., Chen Y., Hao S., Peng X., Hu L. (2019). Deep learning for sensor-based activity recognition: A survey. Pattern Recognit. Lett..

[13] Ordóñez F.J., Roggen D. (2016). Deep convolutional and lstm recurrent neural networks for multimodal wearable activity recognition. Sensors.

[14] Gao W., Zhang L., Huang W., Min F., He J., Song A. (2021). Deep neural networks for sensor-based human activity recognition using selective kernel convolution. IEEE Trans. Instrum. Meas..

[15] Tang Y., Zhang L., Wu H., He J., Song A. (2022). Dual-branch interactive networks on multichannel time series for human activity recognition. IEEE J. Biomed. Health Inform..

[16] Vaswani A., Shazeer N., Parmar N., Uszkoreit J., Jones L., Gomez A.N., Kaiser Ł., Polosukhin I. Attention is all you need. Proceedings of the 31st Conference on Neural Information Processing Systems (NIPS 2017).

[17] Foumani N.M., Tan C.W., Webb G.I., Salehi M. (2023). Improving Position Encoding of Transformers for Multivariate Time Series Classification. arXiv.

[18] Chen C., Wu Y., Dai Q., Zhou H.Y., Xu M., Yang S., Han X., Yu Y. (2024). A Survey on Graph Neural Networks and Graph Transformers in Computer Vision: A Task-Oriented Perspective. IEEE Trans. Pattern Anal. Mach. Intell..

[19] Kang J., Poria S., Herremans D. (2024). Video2Music: Suitable music generation from videos using an Affective Multimodal Transformer model. Expert Syst. Appl..

[20] Shavit Y., Klein I. (2021). Boosting inertial-based human activity recognition with transformers. IEEE Access.

[21] Gao W., Zhang L., Teng Q., He J., Wu H. (2021). DanHAR: Dual attention network for multimodal human activity recognition using wearable sensors. Appl. Soft Comput..

[22] Shaw P., Uszkoreit J., Vaswani A. (2018). Self-attention with relative position representations. arXiv.

[23] Zhang Z., Wang W., An A., Qin Y., Yang F. (2023). A human activity recognition method using wearable sensors based on convtransformer model. Evol. Syst..

[24] Haroon S., Hafsath C., Jereesh A. (2023). Generative Pre-trained Transformer (GPT) based model with relative attention for de novo drug design. Comput. Biol. Chem..

[25] Dosovitskiy A., Beyer L., Kolesnikov A., Weissenborn D., Zhai X., Unterthiner T., Dehghani M., Minderer M., Heigold G., Gelly S. (2020). An image is worth 16x16 words: Transformers for image recognition at scale. arXiv.

[26] Sikder N., Nahid A.A. (2021). KU-HAR: An open dataset for heterogeneous human activity recognition. Pattern Recognit. Lett..

[27] Micucci D., Mobilio M., Napoletano P. (2017). Unimib shar: A dataset for human activity recognition using acceleration data from smartphones. Appl. Sci..

[28] Zhang M., Sawchuk A.A. USC-HAD: A daily activity dataset for ubiquitous activity recognition using wearable sensors. Proceedings of the 2012 ACM Conference on Ubiquitous Computing.

[29] Sejdić E., Djurović I., Jiang J. (2009). Time–frequency feature representation using energy concentration: An overview of recent advances. Digit. Signal Process..

[30] Veiga J.J.D., O’Reilly M., Whelan D., Caulfield B., Ward T.E. (2017). Feature-free activity classification of inertial sensor data with machine vision techniques: Method, development, and evaluation. JMIR mHealth uHealth.

[31] Ronao C.A., Cho S.B. (2016). Human activity recognition with smartphone sensors using deep learning neural networks. Expert Syst. Appl..

[32] Ignatov A. (2018). Real-time human activity recognition from accelerometer data using Convolutional Neural Networks. Appl. Soft Comput..

[33] Zhang C., Cao K., Lu L., Deng T. (2022). A multi-scale feature extraction fusion model for human activity recognition. Sci. Rep..

[34] Tang Y., Zhang L., Min F., He J. (2022). Multiscale deep feature learning for human activity recognition using wearable sensors. IEEE Trans. Ind. Electron..

[35] Guan Y., Plötz T. (2017). Ensembles of deep lstm learners for activity recognition using wearables. Proc. ACM Interact. Mob. Wearable Ubiquitous Technol..

[36] Zhao Y., Yang R., Chevalier G., Xu X., Zhang Z. (2018). Deep residual bidir-LSTM for human activity recognition using wearable sensors. Math. Probl. Eng..

[37] Alawneh L., Mohsen B., Al-Zinati M., Shatnawi A., Al-Ayyoub M. A comparison of unidirectional and bidirectional lstm networks for human activity recognition. Proceedings of the 2020 IEEE International Conference on Pervasive Computing and Communications Workshops (PerCom Workshops).

[38] Hammerla N.Y., Halloran S., Plötz T. (2016). Deep, convolutional, and recurrent models for human activity recognition using wearables. arXiv.

[39] Liu Z., Lin Y., Cao Y., Hu H., Wei Y., Zhang Z., Lin S., Guo B. Swin transformer: Hierarchical vision transformer using shifted windows. Proceedings of the IEEE/CVF International Conference on Computer Vision.

[40] Dirgová Luptáková I., Kubovčík M., Pospíchal J. (2022). Wearable sensor-based human activity recognition with transformer model. Sensors.

[41] Huang Z., Liang D., Xu P., Xiang B. (2020). Improve transformer models with better relative position embeddings. arXiv.

[42] Dai Z., Liu H., Le Q.V., Tan M. (2021). Coatnet: Marrying convolution and attention for all data sizes. Adv. Neural Inf. Process. Syst..

[43] Luong M.T., Pham H., Manning C.D. (2015). Effective approaches to attention-based neural machine translation. arXiv.

[44] Karantonis D., Narayanan M., Mathie M., Lovell N., Celler B. (2006). Implementation of a real-time human movement classifier using a triaxial accelerometer for ambulatory monitoring. IEEE Trans. Inf. Technol. Biomed..

[45] Banos O., Galvez J.M., Damas M., Pomares H., Rojas I. (2014). Window size impact in human activity recognition. Sensors.

[46] Jameer S., Syed H. (2023). Deep SE-BiLSTM with IFPOA Fine-Tuning for Human Activity Recognition Using Mobile and Wearable Sensors. Sensors.

[47] Al-Qaness M.A., Helmi A.M., Dahou A., Elaziz M.A. (2022). The applications of metaheuristics for human activity recognition and fall detection using wearable sensors: A comprehensive analysis. Biosensors.

[48] Teng Q., Zhang L., Tang Y., Song S., Wang X., He J. (2021). Block-wise training residual networks on multi-channel time series for human activity recognition. IEEE Sens. J..

[49] Kumar P., Suresh S. (2022). DeepTransHHAR: Inter-subjects Heterogeneous Activity Recognition Approach in the Non-identical Environment Using Wearable Sensors. Natl. Acad. Sci. Lett..

[50] Cheng X., Zhang L., Tang Y., Liu Y., Wu H., He J. (2022). Real-time human activity recognition using conditionally parametrized convolutions on mobile and wearable devices. IEEE Sens. J..

[51] Essa E., Abdelmaksoud I.R. (2023). Temporal-channel convolution with self-attention network for human activity recognition using wearable sensors. Knowl.-Based Syst..

[52] Abid M.H., Nahid A.A., Islam M.R., Mahmud M.P. Human Activity Recognition Based on Wavelet-Based Features along with Feature Prioritization. Proceedings of the 2021 IEEE 6th International Conference on Computing, Communication and Automation (ICCCA).

[53] Alsarhan T., Alawneh L., Al-Zinati M., Al-Ayyoub M. Bidirectional gated recurrent units for human activity recognition using accelerometer data. Proceedings of the 2019 IEEE SENSORS.

[54] Tahir S.B.U.D., Jalal A., Kim K. (2020). Wearable inertial sensors for daily activity analysis based on Adam optimization and the maximum entropy Markov model. Entropy.

[55] Wieland C., Pankratius V. TinyGraphHAR: Enhancing Human Activity Recognition With Graph Neural Networks. Proceedings of the 2023 IEEE World AI IoT Congress (AIIoT).

[56] Nooyimsai L., Pakdeepong O., Chatchawalvoradech S., Phiakhan T., Laitrakun S. Smartphone-Based Human Activity and Fall Recognition Using Deep Feature Extraction and Machine-Learning Classifiers. Proceedings of the 2022 17th International Joint Symposium on Artificial Intelligence and Natural Language Processing (iSAI-NLP).

[57] Bi H., Perello-Nieto M., Santos-Rodriguez R., Flach P. (2020). Human activity recognition based on dynamic active learning. IEEE J. Biomed. Health Inform..

[58] Khan D., Al Mudawi N., Abdelhaq M., Alazeb A., Alotaibi S.S., Algarni A., Jalal A. (2024). A Wearable Inertial Sensor Approach for Locomotion and Localization Recognition on Physical Activity. Sensors.

[59] Cheng D., Zhang L., Bu C., Wu H., Song A. (2023). Learning hierarchical time series data augmentation invariances via contrastive supervision for human activity recognition. Knowl.-Based Syst..

[60] Montaha S., Azam S., Rafid A.R.H., Hasan M.Z., Karim A., Islam A. (2022). Timedistributed-cnn-lstm: A hybrid approach combining cnn and lstm to classify brain tumor on 3d mri scans performing ablation study. IEEE Access.

[61] Meena T., Sarawadekar K. (2024). An eXplainable Self-Attention-Based Spatial–Temporal Analysis for Human Activity Recognition. IEEE Sens. J..

[62] Liu Z., Luo S., Li W., Lu J., Wu Y., Sun S., Li C., Yang L. (2020). Convtransformer: A convolutional transformer network for video frame synthesis. arXiv.

